# Mn(II) Quinoline Complex (4QMn) Restores Proteostasis and Reduces Toxicity in Experimental Models of Huntington’s Disease

**DOI:** 10.3390/ijms23168936

**Published:** 2022-08-11

**Authors:** Marián Merino, María Dolores Sequedo, Ana Virginia Sánchez-Sánchez, Mª Paz Clares, Enrique García-España, Rafael P. Vázquez-Manrique, José L. Mullor

**Affiliations:** 1 Bionos Biotech SL, Biopolo Hospital La Fe, 46026 Valencia, Spain; 2 Laboratory of Molecular, Cellular and Genomic Biomedicine, Instituto de Investigación Sanitaria La Fe, 46026 Valencia, Spain; 3 Departamento de Química Orgánica e Inorgánica, Instituto de Ciencia Molecular, Universidad de Valencia, 46980 Valencia, Spain; 4 Centro de Investigación Biomédica en Red de Enfermedades Raras (CIBERER), 28029 Madrid, Spain; 5 Joint Unit for Rare Diseases IIS La Fe-CIPF, 46012 Valencia, Spain

**Keywords:** Mn(II) complexes, Huntington’s disease, polyQ toxicity, autophagy, proteasome, *Caenorhabditis elegans*

## Abstract

Huntington’s disease (HD) is an autosomal dominant neurodegenerative disorder, of the so-called minority diseases, due to its low prevalence. It is caused by an abnormally long track of glutamines (polyQs) in mutant huntingtin (mHtt), which makes the protein toxic and prone to aggregation. Many pathways of clearance of badly-folded proteins are disrupted in neurons of patients with HD. In this work, we show that one Mn(II) quinone complex (4QMn), designed to work as an artificial superoxide dismutase, is able to activate both the ubiquitin-proteasome system and the autophagy pathway in vitro and in vivo models of HD. Activation of these pathways degrades mHtt and other protein-containing polyQs, which restores proteostasis in these models. Hence, we propose 4QMn as a potential drug to develop a therapy to treat HD.

## 1. Introduction

Huntington’s disease (HD) is a neurodegenerative disorder caused by an abnormally long expansion of CAG repeats, in exon 1, in the gene encoding for the huntingtin protein (Htt). This expansion encodes a track of glutamines (polyQs) that, when it equals or exceeds 36, makes Htt mutant (mHtt). These abnormally expanded polyQs induce erroneous folding of the protein, making mHtt prone to aggregation due to the cytoplasmic exposure of some hydrophobic domains [1]. mHtt aggregates sequester other proteins, disrupting their function and further exacerbating cellular toxicity. Although mHtt is expressed ubiquitously [2], toxicity affects primarily the striatum and the cortex in the brain of patients and murine animal models of HD, in which neurons are impaired and eventually die [3]. This neuronal toxicity disrupts the striatum and causes HD patients to suffer chorea and limb incoordination, psychiatric impairment, and cognitive deterioration as a consequence of cortex dysfunction.

The function of the Htt protein is under debate as many roles have been attributed to it, including its involvement in the dynamics of the cytoskeleton, gene expression, or neuronal survival [4]. Among these functions, Htt also works to regulate macroautophagy (autophagy from now on) [5]. In this regard, patients and models of HD show reduced autophagy capacity, which in turn results in augmented proteotoxicity as autophagy is one of the main sources of mHtt clearance [6,7]. Apart from autophagy, the ubiquitin-proteasome system (UPS) has also been shown to play a part in removing mHtt from cells, and disruption of this function seems to have a role in the progress of HD [8,9,10]. Therefore, restoring autophagy and proteasome flux using specific inhibitors has shown to be a key therapeutic strategy for fighting HD [2,11,12,13,14].

One characteristic trait of neurodegenerative disorders is the participation of oxidative stress in the progression of the disease [15]. Several strategies to fight HD have been implemented using antioxidants as therapeutic drugs [16]. To counteract oxidative stress in HD, some organic compounds that emulate superoxide dismutase (SOD) have been synthesised [17,18,19]. The structure of these chemicals is such that they are able to carry metal ions, such as manganese, for example, and therefore mimic the SOD catalytic core [17].

In this work, we have assessed the potential of 4QMn, a substance that mimics the active centre of SOD and hence, its antioxidant capacity [17], to alleviate symptoms of HD in vitro and in vivo models. Interestingly, we observed that this substance was able to reduce mHtt and polyQ aggregates. Hence, we tested the capacity of the compound to eliminate mHtt from human cells and *C. elegans* nematodes and restore the functionality of neurons. Moreover, we showed that 4QMn was able to induce the activity of UPS and autophagy in cultured cells, which explains why it rescues proteostasis in worms and human cell models of HD. Therefore, this compound holds promise as a potential therapeutic agent for HD and other neurodegenerative diseases where protein aggregates are involved.

## 2. Results and Discussion

### 2.1. Treating In Vitro and In Vivo Models of HD with 4QMn Reduces Protein Aggregation

**Figure 1:**
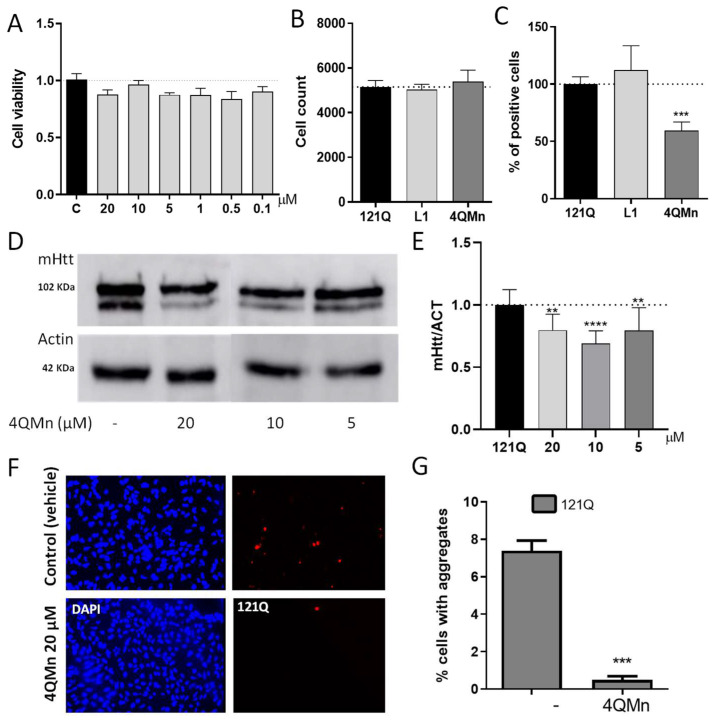
Treating cells expressing mHtt with 4QMn reduces mHtt. (**A**) Cell viability analysis of 4QMn in HEK293 cells. (**B**) Cell count analysis by In-Cell analyzer. (**C**) Percentage of positive cells with mHtt. (**D**) Western Blot of proteins from HEK293 cells transfected with 121Q, and treated with different amounts of 4QMn. (**E**) Quantification of the mHtt from the Western Blot. The two bands most likely represent proteolytic cleavage of mHtt, as suggested elsewhere [24]. (**F**) Representative images of fluorescence taken from one of the experiments of the transfection of 121Q treated (4QMn 20 µM) and non-treated (control-vehicle). (**G**) Quantification of the number of cells showing inclusion bodies from 121Q. ANOVA test with post hoc Tukey. **** *p* < 0.0001, *** *p* < 0.001, ** *p* < 0.01. All experiments were performed three times, at least.

As mentioned above, mHtt acquires an abnormal protein conformation, which leads to aggregation and subsequent cell toxicity [20]. As the aggregation of mHtt is a common feature among the different models of HD, this is the first therapeutic target under study during compound screenings [21]. Therefore, we aimed to determine the ability of 4QMn to reduce total mHtt in human cells. HEK293T cells were transfected with the 121Q plasmid that overexpressed the mHTT_1-586_ fragment of huntingtin and fused in-frame to mCherry. This species of mHtt is naturally released by digestion of the full-length mHtt, by caspase C6, in patients with HD [22]. Moreover, this form of mHtt is believed to be particularly toxic and crucial for the progression of the pathology [22]. Hence, after transfection, the cells were treated with different non-toxic concentrations of 4QMn. These were chosen after performing a cell death assay (MTT assay Figure 1A). After incubation with the compound, the cells were processed for mHtt quantification using immunofluorescence (under an In-Cell Analyzer automatic fluorescence microscope), and in parallel, the cells were lysed, and the total mHtt was analysed by Western blot. In the immunofluorescence assay, quantification was performed with the In-cell Analyzer software, and the cells were also treated with L1 (3,6,9-triaza-1(2,6)-pyridinecyclodecaphane-14-carboxylic acid), a compound that forms Cu^2+^ complexes and mimics SOD activity such as 4QMn [23]. After a 24 h treatment with 4QMn, we observed that the percentage of positive cells with mHtt was significantly decreased by 40.4 ± 8.6%, compared to non-treated cells. However, this effect was not observed when the cells were treated with the L1 compound. This result suggests that the reduction in mHtt protein by 4QMn was not due to its antioxidant functionality as a compound with similar antioxidant capacity, L1, did not reduce mHtt levels (Figure 1B,C).

Additionally, the Western blot analysis also showed that different concentrations of 4QMn (20 µM, 10 µM, and 5 µM) reduced overall mHtt levels when compared to untreated control cells. The Western bands were semi-quantified using image analysis software to obtain approximate values of inhibition of 20.5 ± 6.0%, 31.0 ± 6.0%, and 20.31 ± 6.0%, respectively (Figure 1D,E).

Since treatment with 4QMn reduced the presence of total mHtt, we investigated whether this translated into a reduction in mHtt aggregation. Hence, we took advantage of the presence of mCherry fused in frame with mHtt to quantify protein aggregates using fluorescence microscopy. This analysis showed that 4QMn was also able to substantially reduce the number of cells carrying inclusion bodies, which is an indirect way of measuring mHtt aggregation (Figure 1F,G).

**Figure 2:**
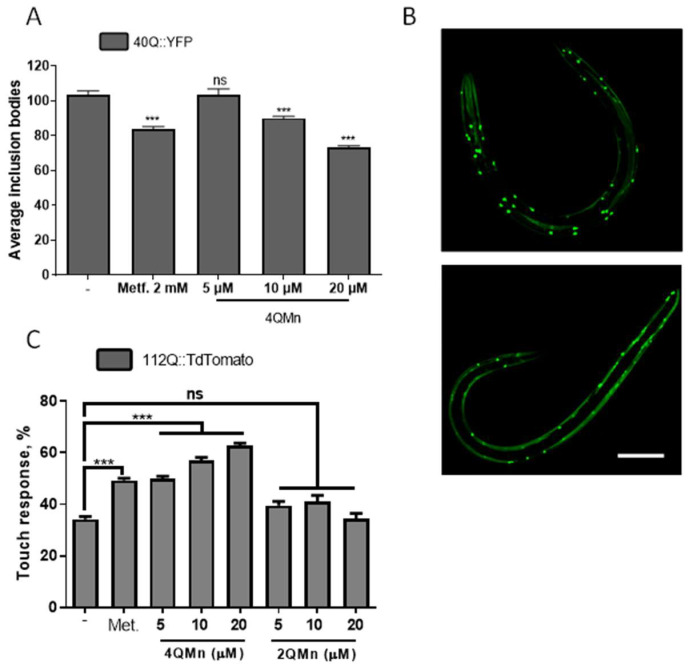
The 4QMn compound reduces polyQ aggregation and reduces toxicity from mHtt in *C. elegans*. (**A**) Growing *C. elegans* worms in different concentrations of 4QMn reduce polyQ aggregation in muscle cells. (**B**) Representative fluorescent images of control (upper image) and 4QMn-treated worms (below). (**C**) Incubating worms with 4QMn rescues neuronal function in worms stressed with mHtt. The antioxidant 2QMn does not induce any effect in worms. A 2 mM metformin (Metf.) was used as a positive control for polyQ aggregation reduction and alleviation of toxicity induced by mHtt. The worms were synchronized and incubated with the drug from the L1 stage. The animals were tested when they were at the young adult stage. ANOVA test with post hoc Tukey. *** *p* < 0.001; ns non-statistically significant. Bar 0.1 mm.

Since the 4QMn was able to reduce mHtt aggregation in vitro, we tested whether this compound was also able to alleviate phenotypes in vivo. To do so, we used *C. elegans* expressing polyQs (40Q::YFP) in muscle cells [25]. These animals allow for the investigation of the dynamics of polyQ aggregation because polyQ peptides collapse and produce inclusion bodies that are easily observed and quantified using a dissecting fluorescence microscope [13,14,25]. Additionally, these 40Q:YFP worms also show signs of oxidative stress [26]. We observed that treating these animals with different concentrations of the 4QMn compound resulted in a statistically significant, dose-dependent reduction in inclusion bodies (Figure 2A,B). In this model of polyQ toxicity, exacerbating the presence of free radicals or reducing oxidative stress by means of antioxidants does not affect the aggregation dynamics [26]. Hence, we hypothesised that the 4QMn compound might be acting through different pathways of protein clearance.

In order to confirm that a reduction in polyQ aggregation by treatment with 4QMn translated into an improvement of the disease phenotype, we tested neuronal function. We used animals expressing the first exon of huntingtin fused in frame with TdTomato in mechanosensory neurons [14]. An expression of this construct induces neuronal impairment that can be assayed using the “touch assay”, which measures the ability of the mechanosensory neurons to feel light mechanosensation [27]. This assay has been widely used before to test treatments in worm models of HD [27,28,29,30]. Functional analysis of the mechanosensory neurons of the animals expressing 112Q, treated with 4QMn, showed that this compound was able to restore neuronal function, which suggests that this substance has therapeutic potential to treat HD (Figure 2C). As described above, the 4QMn compound mimics the SOD enzyme active centre and acts as an antioxidant molecule. Hence, the rescue of the neural function might be due to a reduction in oxidative stress. To explore this hypothesis, we treated the animals expressing 112Q with 2QMn, another Mn SOD mimic molecule, which has the same molecular formula (C23H3ON6) as 4QMn, where the quinone is bound to C2 (2QMn) instead of C4 (4QMn) [17,18]. However, treatment with 2QMn did not restore the neuronal function in worms expressing 112Q in mechanosensory neurons (Figure 2C). This suggests that antioxidant function was not sufficient for neural function recovery. Moreover, these results suggested that the beneficial effect of 4QMn was exclusively due to the induction of other pathways or cellular processes. In this regard, it has been described before that reduction in oxidative stress in diverse experimental models as well as in patients affected by HD did not necessarily translate into the functional recovery of a given phenotype (recently reviewed by Bono-Yagüe et al., 2020) [16].

### 2.2. The Proteasomal System Is Impaired by mHtt and Treatment with 4QMn Restores Its Activity

**Figure 3:**
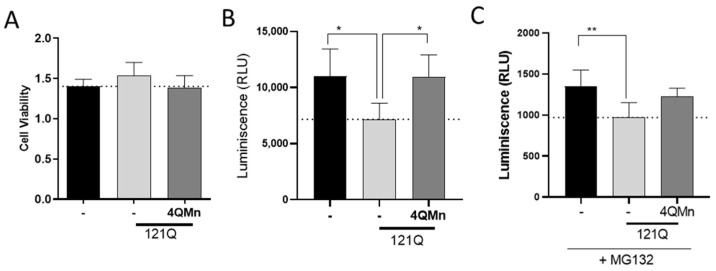
Treating mHtt-stressed cells with 4QMn reduces toxicity, and this effect is partially UPS-independent. (**A**) Cell viability data (MTT assay). (**B**) Graph showing proteasomal activity, measured by luminescence, in naïve HEK293 cells, cells transfected by mHtt, and cells transfected and treated with 4QMn. (**C**) The same data, but with all treatments with the proteasome inhibitor MG132. * *p* < 0.05; ** *p* < 0.01; ANOVA test with post hoc Tukey.

The UPS pathway is one of the most important pathways for the degradation of toxic proteins in eukaryotic cells. In models of polyQ-induced toxicity, including HD [9,10], UPS substrates accumulate throughout the cell and induce toxicity through direct impairment of proteasomal degradation [31]. Several strategies to develop therapies to treat HD have relayed into activation of the UPS pathway [32,33,34,35,36,37]. Therefore, we tested the potential of 4QMn as a proteasome activator in our HD cell model. To do so, we transfected HEK293T cells with the plasmid that expresses the mHtt isoform with the N-terminal 585 first amino acids, carrying 121Q. Then, we incubated the cells with 4QMn for 24 h. An analysis of the proteasomal activity showed that the cells expressing mHtt had significantly reduced their proteasomal activity by 38.3% (±12.5%) without affecting cell viability compared to control cells (Figure 3A,B), a result that is in agreement with the literature [9,38]. On the other hand, treatment with 4QMn reduced proteasomal activity to the same level as control cells (Figure 3A,B). To further investigate this, we used MG132, a well-known proteasome inhibitor. MG132 reduced proteasome activity in the control cells as luminescence was one order of magnitude lower (Figure 3C) compared to our experiment without inhibitor (Figure 3B). As expected, mHtt further reduced proteasome activity in MG132 treated cells (Figure 3B). In this case, incubation with 4QMn showed a trend to rescue proteasomal activity, although it was not statistically significant. Altogether, these data show that the mechanism of action of 4QMn is clearly related to the pathways of protein degradation.

### 2.3. 4QMn Promotes Autophagy in Cells Stressed by mHtt

Autophagy is a conserved and essential cell process that allows the degradation of damaged intracellular components, such as organelles, misfolded proteins, and foreign bodies [39]. The maintenance of normal cellular proteostasis primarily depends on autophagy. In fact, altered autophagy causes protein aggregation, and this is one of the hallmarks of HD and other neurodegenerative diseases [40]. Numerous studies have demonstrated that mHtt protein is an autophagy substrate (reviewed by Valionyte et al. [7]). Moreover, activation of autophagy in models of HD increased mHtt clearance and cell survival [41,42,43,44]. Interestingly, wild-type Htt is a component of the pathway that is required for selective degradation [5]. Thus, autophagy modulation has been proposed as a therapeutic intervention in HD [14,30,45]. We tested autophagy in our in vitro model of HD and the effect of 4QMn on autophagy. We monitored the autophagy flux by studying different auto-phagosomal membrane markers or cargo-adaptors. These biomarkers are the microtubule-associated protein LC3 and p62/SQSTM1 (p62). LC3I is a soluble protein that is conjugated with phosphatidylethanolamine to become LC3II, which is located in both the outer and inner membranes of auto-phagosomes [46]. When autophagy is induced, the number of auto-phagosomes and auto-phagosomal carrying LC3II is increased. However, as soon as the auto-phagosome fuses with the auto-lysosome, degradation of LC3II occurs and a decrease in this protein can be assessed [47]. For this purpose, it is absolutely necessary to use a lysosome-specific inhibitor, such as bafilomycin A_1_ (Baf A_1_), that blocks the fusion between auto-phagosome and auto-lysosome, enabling LC3II accumulation and quantification. On the other hand, degradation of p62 is another marker widely used to monitor autophagy as it directly binds to LC3 and is degraded by autophagy selectively [48]. The p62 protein is a ubiquitin-binding scaffold protein, also called sequestosome 1 (SQSTM1), that colocalizes with ubiquitinated proteins in several neurodegenerative diseases such as HD [49]. In the same way as for LC3II, it is rapidly degraded when the fusion with the auto-lysosome occurs. Thus, using baf A_1_ is also mandatory to be able to quantify the levels of autophagy induction.

**Figure 4:**
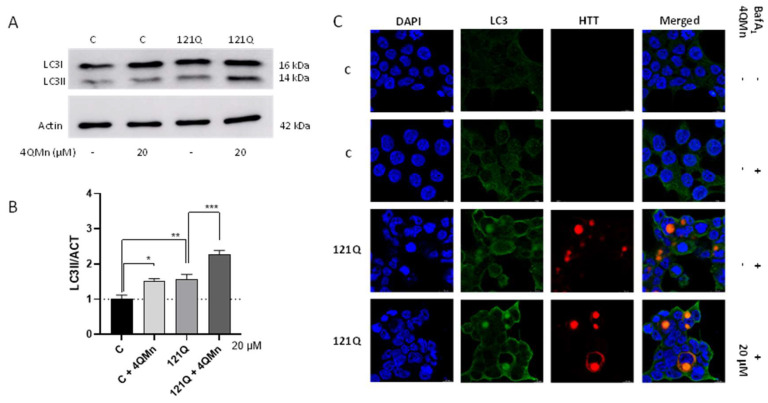
The compound 4QMn induces autophagy in cells stressed by mHtt. (**A**) Western blot analysis of the expression of the LC3 forms. Actin was detected to perform normalization of the samples. (**B**) Graph with the quantification of the western blot from A. (**C**) Fluorescence imaging of the cultures of HEK293 naïve and transfected with mHtt, stained for nuclei (blue), mHtt (mCherry/red), and LC3 (green). ANOVA test with post hoc Tukey. * *p* < 0.05; ** *p* < 0.01; *** *p* < 0.001.

The Western blot analysis revealed that 20 µM 4QMn significantly increased LC3II protein levels in naïve cells (without mHtt), which strongly suggests that this substance is able to potently activate autophagy (Figure 4A,B). On the other hand, there were also significant differences between the control cells and cells expressing mHtt and treated with 4QMn (Figure 4A,B). In addition, the LC3II levels were increased in this HD cell model, as has been observed in previous studies [43,50,51,52]. Finally, the treatment with 4QMn also significantly increased LC3II in cells with mHtt aggregates (Figure 4C), which further suggests that the autophagy flux was activated. Regarding p62, Western blot analysis did not show any significant difference among the conditions tested (data not shown).

### 2.4. 4QMn Activates UPS and Autophagy for mHtt Degradation

**Figure 5:**
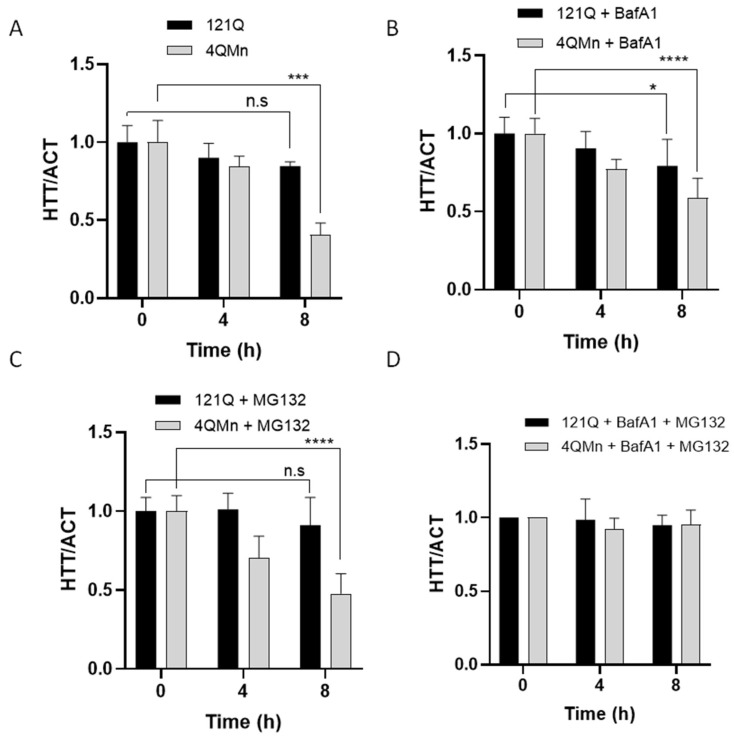
mHtt degradation is UPS- and autophagy-dependent, and 4QMn induces both pathways. (**A**–**D**) Graphs describing the data on 4QMn-induced degradation of mHtt, over time, and negative control (vehicle). (**A**) Without including any drug treatment. (**B**) With the autophagy inhibitor bafilomycin A1 (BafA1). (**C**) With the UPS inhibitor MG132. (**D**) With both inhibitors, Bafilomycin A1 and MG132. Ns: non-statistically significant; * *p* < 0.05; *** *p* < 0.001; **** *p* < 0.0001, ANOVA test, with post hoc Tukey.

It is well-known that mHtt aggregates accumulate in the cytoplasm, causing the loss of normal physiological functions and the gain of toxic functions [4,53]. We have shown above that 4QMn activates both the UPS and autophagy pathways to remove mHtt aggregates. Hence, we sought to investigate the mechanism of action of this compound. To this end, the degradation rate of mHtt was studied using the cycloheximide (Chx) chase assay under the effect of the proteasomal inhibitor, MG132, the lysosome inhibitor, baf A_1_ or both compounds simultaneously. Through performing the Western blot image analysis, mHtt was quantified over time in the presence of Chx and the effect of 4QMn was evaluated. The results showed that 4QMn treatment induced a substantial degradation (59.5 ± 0.1%) of mHtt after 8 h (Figure 5A). When the lysosomal inhibitor baf A_1_ was added to the Chx treated cells, the 4QMn treatment still reduced mHtt degradation, although at lower levels (Figure 5B). Similarly, when the proteasome inhibitor MG132 was added in the presence of Chx, the 4QMn treatment still induced a significant decrease in mHtt levels (Figure 5C), suggesting that 4QMn still exerted its action. Finally, both inhibitors, MG132 and baf A_1_, were added along with 4QMn. This treatment resulted in the complete inhibition of 4QMn function, and mHtt degradation was not induced (Figure 5D). These results clearly demonstrate that 4QMn induces mHtt degradation through both the UPS and autophagy pathways, and blockade in one of the pathways can be overcome by activation of the other as mHtt degradation was induced at similar levels as in control cells.

Future strategies to delay the progression of HD—and maybe other neurodegenerative diseases—may consist of a combination of small molecules that synergise with more complex therapies in the degradation of mHtt, and hence alleviate the symptoms of patients with HD. Some of these strategies include genetic means, such as silencing the expression of the gene using RNAi, which induces degradation of the messenger and then encodes the protein [54]. Other ways to do so are the use of antisense oligonucleotides (ASO) to block the translation of this messenger. ASOs are currently being used in clinical trials [54,55,56,57,58,59,60,61,62,63,64]. Some strategies that are under investigation involve gene targeting to edit HTT, using CRISPR, for example, to remove the CAG expansion from the gene [65]. However, these techniques are in their infancy, require further research to make them useful, and even they would require specialized personnel to deliver therapies and genome modification in humans [66]. Most of these techniques and strategies are reviewed by Tabrizi and collaborators [67].

Some small compounds have shown the potential to reduce the expression of mHtt. This, in contrast to DNA delivery techniques, allows for systemic elimination of the toxic cause of HD, mHtt. These small molecules, compared to DNA or single-stranded Oligodeoxynucleotides (ssODNs), can reach the nervous system and most organs, can be easily administered (orally) and would be widely available to all patients without the need to attend specialized delivery centres. Systemic administration is also important because Htt is ubiquitously expressed, and many non-neuronal phenotypes have also been described so far [2,4]. Therefore, the 4QMn molecule could be a potential candidate for the treatment of HD and maybe other neurodegenerative diseases.

## 3. Materials and Methods

### 3.1. Cell Culture and Transfection

Human embryonic kidney 293T cells (HEK293T) were cultured in Gibco DMEM medium, low glucose (Fisher scientific, Madrid, Spain) supplemented with L-Glutamine (Sigma Aldrich, Darmstadt, Germany), penicillin-streptomycin (Gibco, Fisher Scientific, Madrid, Spain) and fetal bovine serum (Gibco, Fisher Scientific, Madrid, Spain). Cells were harvested with trypsin/EDTA (Gibco, Fisher Scientific, Madrid, Spain) when 80% confluence was reached, and they were seeded at a density of 8 × 10^3^ cells/mL into 96-well plates or 50 × 10^3^ cells/mL into 24-well plates (Corning Incorporated, Kennenbunk, ME, USA). Cultures were maintained at 37 °C in a humidified incubator supplying 5% CO_2_/air. For generating the Huntington’s disease cell model, the transient expression of mHtt was induced. HEK293T cells were transfected using Lipofectamine 3000 (Invitrogen, Fisher Scientific, Madrid, Spain), and the complexes were prepared and incubated according to the manufacturer’s protocol using Opti-MEM reduced serum medium (Fisher Scientific, Madrid, Spain). The plasmid used for the overexpression of huntingtin protein contained the huntingtin exon 1 with a polyQ stretch of 121 glutamines in frame with the red fluorescent protein mCherry and the CMV promoter. After 24 h incubation, the transfection reagent was removed, and cells were treated with 4QMn compound at 5–10–20 µM concentrations to evaluate its efficacy in the subsequent assays (three biological replicates). The concentrations used in these experiments were not cytotoxic, previously determined by the MTT assay.

### 3.2. Worm Culture and Manipulation

*C. elegans* worms were maintained and assayed at 20 °C, as described elsewhere [68]. The 40Q model [25] was obtained from the repository of worms Caenorhabditis Genetics Center (https://cgc.umn.edu/ (accessed on 20 June 2022)). The 112Q model was created by Sanchis et al. [14]. The collection of polyQ aggregation from 40Q animals was performed on life worms while foraging in Petri dishes, using a dissecting microscope equipped with fluorescence (Leica TCS SP5-AOBS, headquarters), following the procedures described elsewhere [13,14]. The mechanosensation data was collected using an eyelash mounted on a toothpick, following procedures described elsewhere [13,14,29,30,55]. We performed at least three independent experiments per type of assay. The worms were synchronized and incubated with the drug from the L1 stage. The animals were tested when they were in the young adult stage. The worms were cultured in liquid with the 4QMn or vehicle (controls) on a shaker as described elsewhere [13,14]. An ANOVA test was used to investigate statistical significance, and the post hoc of Tukey was applied to find out each *p*-value.

### 3.3. MTT Assay

To evaluate cell viability, 3-(4,5-dimethylthiazol-2-yl)-2,5-diphenyltetrazolium bromide (Sigma Aldrich, Darmstadt, Germany) was used. HEK293 cells treated with the different compounds were incubated with the MTT reagent for 3 h. After the incubation period, dimethyl sulfoxide (Sigma Aldrich, Darmstadt, Germany) was used to dissolve the formazan, and the colour intensity was measured at 550 nm in a spectrophotometer Halo LED 96 (Dynamica Scientific, Livingston, UK).

### 3.4. Immunofluorescence Quantification through In-Cell Analyzer

For protein quantification by immunofluorescence analysis, the In-Cell Analyzer 2000 (GE Healthcare Life Sciences, Chicago, IL, USA) was employed. After the transfection, cells were incubated for 24 h in a medium (control) or medium containing 5 µM of each compound. Following that, cells were processed for microscope analysis. First, cells were fixed with paraformaldehyde 2% (Electron Microscopy Sciences, Hatfield, PA, USA) in DMEM medium for 15 min. Then, they were stained with DAPI and Phalloidin 488 (Sigma Aldrich, St. Louis, MO, USA) for 30 min. After that, cells were washed and kept in PBS (Gibco, Sigma Aldrich, St. Louis, MO, USA) for image analysis. The In-Cell Investigator image analysis software was employed to determine the percentage of cells with huntingtin (positive cells) and the percentage of cells without (negative cells).

### 3.5. Western Blot Analysis

For protein quantification by Western blot, transfected cells were washed with PBS buffer (Gibco, Fisher Scientific, Madrid, Spain) before RIPA buffer (150 mM NaCl, 0.1% Triton X-100, 0.5% Sodium Deoxycholate, 0.1% SDS, 2 mM EDTA and 50 mM Tris-HCl, pH 8.0), and a protease inhibitor cocktail (Sigma Aldrich, Darmstadt, Germany) was added to lyse the samples. After that, a cell scraper was performed to detach cells from the well plate, and a Pierce BCA protein assay (Thermo Scientific, Bannockburn, IL, USA) was employed to quantify the number of proteins in the lysate. Protein extracts were separated by 10% or 15% (depending on the size of the protein of interest) sodium dodecyl sulphate-polyacrylamide gel electrophoresis (SDS-PAGE) and transferred to polyvinylidene di-fluoride (PVDF) membranes (Bio-Rad Laboratories, Hercules, CA, USA). After transfer, the membrane was blocked in 5% milk solution with Tween20 0.1% (Sigma Aldrich, Darmstadt, Germany) for 1 h and probed with the following primary antibodies: 1/5000 rabbit monoclonal [EPR5526] to Htt (ab109115), 1/2000 rabbit monoclonal [EPR18709] to LC3B (ab192890), 1/200 mouse monoclonal to SQSTM1/p62 (ab56416), and 1/5000 rabbit monoclonal to beta-Actin (ab8227), all from Abcam, Cambridge, UK. The secondary antibodies used were 1/10,000 goat anti-rabbit IgG H&L HRP (ab97051) and 1/10,000 goat anti-mouse IgG HRP (ab205719), also from Abcam (Cambridge, UK). Chemiluminescent detection was performed using ECL detection kit (Amersham, Fisher Scientific, Madrid, Spain). Densitometry analysis was performed using Image J software (version 1.52, Madrid, Spain).

### 3.6. Proteasome Activity

To measure proteasome activity, the Proteasome-Glo chymotrypsin-like cell-based kit (Promega Biotech Ibérica, Madrid, Spain) was used according to the manufacturer’s instructions. HEK293T cells were seeded in a white 96-well plate, and after transfection, they were treated with 4QMn at 20 µM for 24 h. After the incubation period, cells were processed according to the protocol of the kit. When the activity of the proteasome wanted to be inhibited with a possible rescued capacity of 4QMn, the proteasome inhibitor, MG132 (M7449, Sigma Aldrich, Darmstadt, Germany) was used at 10 µM. In this case, cells were treated with 4QMn and MG132 simultaneously.

### 3.7. Autophagy Markers Study

To quantify autophagy activity, it is necessary to use a lysosome-specific inhibitor, Bafilomycin A_1_ (Baf A_1_). For these studies, after cell transfection and treatment with 4QMn at 20 µM for 24 h, Baf A_1_ is added to the cells at 20 nM during 4 h. After the incubation time, cells are processed for subsequent studies.

### 3.8. Immunostaining

For microscope analysis, cells were fixed with paraformaldehyde 4% (Electron Microscopy Sciences, Hatfield, PA, USA) in PBS for 20 min. Then, cells were washed with PBS and blocked with 10% fetal bovine serum (FBS) (Gibco, Fisher Scientific, Madrid, Spain) in PBS buffer with 0.1% Tween20 for 1 h at room temperature. After that, cells were incubated with primary antibody rabbit monoclonal to LC3B at 1 µg/mL (ab192890) or mouse monoclonal to SQSTM1/p62 at 1/200 (ab56416) in blocking solution overnight, at 4 °C in a humidified chamber. After the incubation period, cells were washed again with PBS, and the secondary antibody Alexa 488 anti-mouse or anti-rabbit 1/200 (Invitrogen, Fisher Scientific, Madrid, Spain) was added to blocking solution with DAPI 1/100 (Invitrogen, Fisher Scientific, Madrid, Spain) for 1 h. Finally, cells were washed and mounted with FluorSave reagent (Calbiochem, Merck Millipore, Darmstadt, Germany).

### 3.9. Cycloheximide Chase Assay (CHX)

The CHX assay was performed to study the degradation kinetics of mHtt. First, HEK293T cells were transfected with the plasmid with 121Q. After 24 h transfection, cells were washed with PBS to remove the transfection reagent, and 4QMn at 20 µM was added together with cycloheximide at 300 µM (Sigma Aldrich, Darmstadt, Germany). Then, cells were washed, and RIPA buffer was added at time 0–4–8 h after the addition of cycloheximide reagent. Afterward, cells were processed for Western blot analysis as described before.

### 3.10. Statistical Analysis

GraphPad Prism software, version 8 (GraphPad, San Diego, CA, USA), was used to perform the statistical analysis. Data are represented as mean ± SD, and the ordinary one-way ANOVA test with Dunnett’s post hoc and Mann–Whitey U test were applied for the analysis. Statistical significance was set at *p* < 0.05, 95% of confidence.

## 4. Conclusions and Final Remarks

In this work, we showed that a small water-soluble molecule, able to cross the blood-brain barrier, can reduce the aggregation of mHtt in human cell models and *C. elegans* transgenic models. Moreover, the 4QMn molecule acts by activating the UPS and the autophagy pathways. These data point to the 4QMn molecule as a potential orally delivered candidate to treat HD systemically. Furthermore, because of the wide roles of protein aggregation in neurodegenerative diseases, the 4QMn molecule may also have applications in other pathologies in which autophagy or proteasome activation may relieve some of the symptoms.

## 5. Patents

4QMn compound synthesis and use are subjected to several international patents.

## Data Availability

Data will be available upon request.
